# Enterprise-wide simultaneous deployment of ambient scribe technology: lessons learned from an academic health system

**DOI:** 10.1093/jamia/ocaf186

**Published:** 2025-10-31

**Authors:** Aileen P Wright, Carolynn K Nall, Jacob J H Franklin, Sara N Horst, Yaa A Kumah-Crystal, Adam T Wright, Dara E Mize

**Affiliations:** Department of Biomedical Informatics, Vanderbilt University Medical Center, Nashville, TN 37203, United States; Department of Medicine, Vanderbilt University Medical Center, Nashville, TN 37232, United States; HealthIT, Vanderbilt University Medical Center, Nashville, TN 37232, United States; Department of Biomedical Informatics, Vanderbilt University Medical Center, Nashville, TN 37203, United States; HealthIT, Vanderbilt University Medical Center, Nashville, TN 37232, United States; Department of Medicine, Vanderbilt University Medical Center, Nashville, TN 37232, United States; HealthIT, Vanderbilt University Medical Center, Nashville, TN 37232, United States; Department of Biomedical Informatics, Vanderbilt University Medical Center, Nashville, TN 37203, United States; HealthIT, Vanderbilt University Medical Center, Nashville, TN 37232, United States; Department of Biomedical Informatics, Vanderbilt University Medical Center, Nashville, TN 37203, United States; Department of Medicine, Vanderbilt University Medical Center, Nashville, TN 37232, United States; HealthIT, Vanderbilt University Medical Center, Nashville, TN 37232, United States; Department of Biomedical Informatics, Vanderbilt University Medical Center, Nashville, TN 37203, United States; Department of Medicine, Vanderbilt University Medical Center, Nashville, TN 37232, United States; HealthIT, Vanderbilt University Medical Center, Nashville, TN 37232, United States

**Keywords:** ambient scribing, clinical documentation, artificial intelligence, electronic health records, burnout

## Abstract

**Objectives:**

To report on the feasibility of a simultaneous, enterprise-wide deployment of EHR-integrated ambient scribe technology across a large academic health system.

**Materials and Methods:**

On January 15, 2025, ambient scribing was made available to over 2400 ambulatory and emergency department clinicians. We tracked utilization rates, technical support needs, and user feedback.

**Results:**

By March 31, 2025, 20.1% of visit notes incorporated ambient scribing, and 1223 clinicians had used ambient scribing. Among 209 respondents (22.1% of 947 surveyed), 90.9% would be disappointed if they lost access to ambient scribing, and 84.7% reported a positive training experience.

**Discussion:**

Enterprise-wide simultaneous deployment combined with a low-barrier training model enabled immediate access for clinicians and reduced administrative burden by concentrating go-live efforts. Support needs were manageable.

**Conclusion:**

Simultaneous enterprise-wide deployment of ambient scribing was feasible and provided immediate access for clinicians.

## Introduction

AI-powered ambient scribe technology was developed to alleviate the burden of clinical documentation, a major identified contributor to clinician burnout.^1^ Studies of ambient scribe implementations have found potential improvements in documentation time, clinician experience, and patient-provider interaction, while also identifying issues with workflow integration, drafted note quality, and financial costs.^2–8^ While one organization^9^ reported on a large pilot of an ambient scribing tool, most studies of EHR-integrated ambient scribing have consisted of small groups of, for example, less than 200 clinicians using ambient scribing. Here we describe the simultaneous deployment of EHR-integrated ambient scribe technology to thousands of clinicians on a single date at a large academic health system. We report on technical support needs, adoption rates, and clinician feedback, and discuss strategies and lessons learned for planning an enterprise-wide implementation of an ambient scribe tool.

## Methods

### Context

Vanderbilt University Medical Center (VUMC) is a large urban academic medical center in Nashville, Tennessee. VUMC sees over 3.2 million patient visits per year in over 180 ambulatory locations. VUMC’s EHR, Epic, was implemented in November 2017. All ambulatory and emergency department (ED) physicians, including resident physicians, and advanced practice providers (APP), but not medical or APP students, were included in this implementation. This project was conducted as an approved quality improvement initiative at VUMC.

### Vendor selection, pilot, and procurement process

After reviewing multiple ambient scribe vendors, with a focus on EHR-integrated solutions, we piloted Nuance’s Dragon Ambient eXperience (DAX), now rebranded as Microsoft Dragon Copilot. The 8-month pilot began in March 2024 with 10 ambulatory users and eventually expanded to include over 54 ambulatory clinicians from multiple specialties and 37 ED clinicians. User feedback was used to refine the tool for our institution. Overall, we received positive feedback from pilot users, and an early analysis of “pajama time” (time spent working on notes outside of scheduled hours) and documentation time suggested benefits of ambient scribing for burden reduction.

### Resource allocation

Following the initial pilot phase, our organization decided to provide all clinicians with access to this tool under an enterprise license, with the objectives of reducing burden, supporting clinician well-being, and improving clinician retention. As such, we did not require providers to pay for the tool or increase their patient load. Approximately 40% of the total cost was offset by eliminating redundant virtual scribe contracts and reducing reliance on in-person scribes.

### Description of technology

At the time of implementation, the ambient scribing tool was compatible with iOS devices (iPhone/iPad), but not Android. After patient consent, providers used the EHR’s mobile app (Haiku; Epic Systems) to record visits. Once the visit ended and recording was stopped, a transcript was sent to the Microsoft cloud. Within minutes, AI-generated documentation was available via dot phrases (ie, for the History of Present Illness, Physical Exam, Results, and Assessment and Plan sections), which populated the note template and could be edited by the provider. Providers could also customize the style of the generated text, such as using a structured or bulleted format.

### Planning for enterprise-wide deployment

#### Implementation team

We formed an implementation team of 10 individuals to support the enterprise-wide deployment. The team included the Chief Medical Information Officer, a project manager, two analysts with expertise in the EHR and ambient documentation tools, three provider-facing EHR support personnel, a trainer focused on curriculum development and clinician education, a data analytics developer, and a clinical informatics fellow.

#### Technical planning

We compiled a list of all eligible users, including physicians and advanced practice providers, from our EHR’s clinical data warehouse. In addition to granting access to clinicians in ambulatory and emergency settings where ambient scribing was being deployed, we also granted access to those in future deployment (eg, inpatient) areas to minimize technical effort and planning as support expanded.

#### Metrics and outcomes

We analyzed utilization data during the study period of January 1, 2025, through March 31, 2025. Metrics included the number of unique users and active users, defined as those who had used the ambient scribe at least once in the past 30 days, looking back to December 1, 2024. We also determined the percentage of notes written using ambient scribing, using as a denominator the total number of ambulatory and ED visit notes, including those written by clinicians who never used ambient scribing during the study period.

#### Technology diffusion

Ahead of go-live, we met with chairs and other departmental leaders to discuss potential benefits, share pilot results and feedback, and outline training opportunities, implementation timelines, and support resources. These leaders then promoted the tool within their departments via emails and meetings. We also raised awareness through institutional publications, clinician champion forums, clinical workstation screensavers, posters, and handouts in clinic areas. We planned a patient-facing announcement in a quarterly patient portal newsletter.

#### Training, support, and monitoring

We developed a self-service website that users accessed for fast, simple training on ambient scribing. On arriving at the website, users could watch a video demonstration of the tool, view step-by-step guidance for getting started, and download instructions on how to set up and use ambient scribing. These instructions covered obtaining patient consent, accessing the ambient scribing tool on an iOS device, and creating ambient scribe-compatible note templates. Importantly, training materials emphasized the need to read and edit the note, since ambient scribing tools can make errors. Users could also view recorded and live webinars, request one-on-one support, or schedule departmental webinars. Training was strongly encouraged but not required. We also planned for in-person support at clinic-area booths and engaged the HealthIT provider support team to be available for additional assistance. In addition, we leveraged existing institutional support systems, including the help desk and incident reporting infrastructure, to monitor user needs and identify any critical incidents following deployment.

#### Post-Implementation survey

We distributed a voluntary survey to all clinicians who had used ambient scribing, 45 days after their first use of ambient scribing. The survey included multiple-choice and free-text questions on documentation quality, efficiency, clinician well-being, and overall experience. We included all surveys sent to clinicians who used the tool before the end of the study period and collected responses through June 15, 2025.

## Results

### Deployment

**Figure 1.**
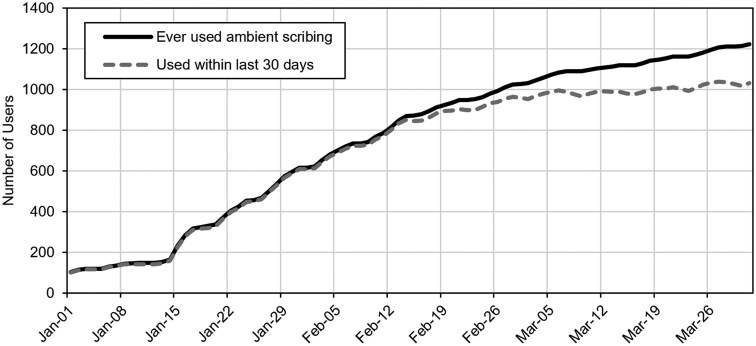
Number of ambient scribing users after enterprise-wide rollout.

**Figure 2.**
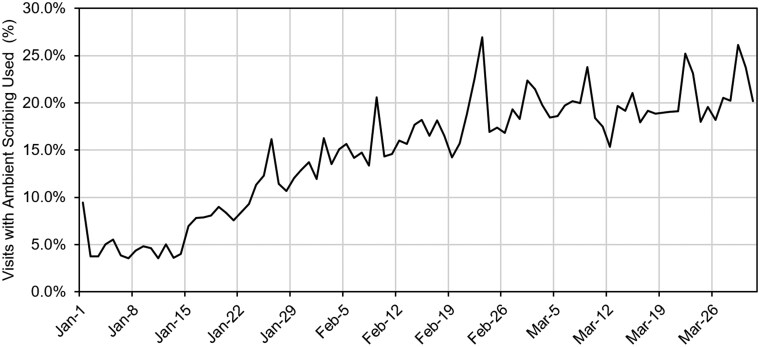
Percent of ambulatory and emergency department visits notes with ambient scribing used.

We deployed ambient scribing across the VUMC enterprise on January 15, 2025, simultaneously turning on access to the ambient scribe technology for over 2400 users. Our utilization trends demonstrated a steady increase in users for the first 4 weeks post-deployment, followed by a period of slower growth (Figure 1), nearing a plateau in active users. On the day of rollout, the number of users increased to 233 and to over 1000 by February 28. At the end of the 3-month study period, there were 1223 users, including 1032 active users who had used ambient scribing at least once in the last 30 days. The percentage of ambulatory and ED visit notes that incorporated ambient scribing increased over time, from 4.2% in the week prior to go-live to 20.1% in the last week of the study period (Figure 2).

### Training, support, and monitoring

During the study period, we gave 24 presentations, including 14 high-level overviews, 7 detailed trainings, and 3 “tips & tricks” sessions to a broad range of clinical and administrative audiences, including medical directors, clinical leadership committees, clinician champions, and grand rounds. We also held 12 webinars and 3 style customization sessions, with a total attendance of 25 participants. Low traffic at staffed go-live support booths led us to pivot to targeted in-clinic “rounding blitzes”—multi-location efforts involving both EHR and ambient scribe support staff—to identify barriers to use and provide real-time assistance. During the study period, 34 ambient scribing-related help desk tickets were submitted, predominantly involving access to the tool, documentation generation, configuration, and training needs. No critical or safety events related to ambient scribing were identified during the study period.

### Survey responses

Surveys were sent to 947 clinicians, of which 209 (22.1%) responded. Of those responding, most (84.7%) rated their training experience positively. Most clinicians (79.0%) reported improved documentation quality, while 9.1% said it worsened. On average, respondents estimated saving 6 minutes per encounter. A majority of survey respondents reported improvements in work-life balance (65.1%), reduced burnout or fatigue (65.1%), were less likely to leave their organization (57.4%), and were less likely to leave clinical practice (57.9%). Overall, most survey respondents said they would be very disappointed (67.0%) or somewhat disappointed (23.9%) if ambient scribing was no longer available. In free-text responses, users expressed enthusiasm about the ambient scribe’s ability to improve patient interaction, save time, enhance work-life balance, and capture more detailed patient histories. Some described the technology as a “game changer,” “life-changing,” and “by far the biggest improvement in my job in the past 10 years.” Others noted that the generated text was often repetitive or included irrelevant or inaccurate information, requiring frequent and substantial edits to the assessment and plan section—sometimes diminishing or negating perceived time savings, particularly in comparison to an effective in-person scribe. Users also requested greater ability to 'teach’ the ambient scribe, customize generated text to their individual documentation style, and adapt the tool to both specialty-specific and user-specific workflows.

## Lessons learned

### Access without added productivity requirements

As we considered various financial models for resourcing ambient scribing at our institution, we ultimately prioritized the goal of investing in our providers’ well-being and enhancing their efficiency. While access to resources such as scribes at other institutions has sometimes been packaged with a requirement to see additional patients per clinic session, we did not impose these requirements for accessing ambient scribing. Survey responses indicated improvements in burnout among those who responded. However, while we report on early results of the deployment, the longer-term ramifications of this investment are not known. As with any institution-wide investment in providers, the broad availability of ambient scribing has the potential to influence future organizational strategies and expectations around clinical efficiency, with potential workload implications for all clinicians, regardless of individual adoption.

### Enterprise-wide access

When faced with the decision to purchase either a limited number of provider licenses or to enter into an enterprise-wide license agreement, we weighed cost savings against equitable access. While some clinician and visit factors may predict who benefits most from the technology, they aren’t definitive, and restricting access could overlook users who might benefit from a low-barrier trial. At academic centers like ours, clinical effort is often fragmented across roles, with many faculty members spending a minority of their time in ambulatory settings. This, combined with rotating trainees, makes targeted licensing difficult and could lead to inadvertent exclusion of part-time or intermittent ambulatory clinicians who still face documentation burdens. An enterprise license avoided these issues, enabling broad access and reducing the burden on IT and administrative teams, who would otherwise need to manage ongoing license allocation and cost tracking.

### Importance of stakeholder engagement

We felt our implementation benefited from early engagement of a small group of enthusiastic clinician champions who became key early adopters and pilot users. We prioritized finding pilot users with established credibility among their peers who were willing to engage and invest time in the pilot, were flexible and open to changing their workflows to adopt the new tool, and were motivated to improve their documentation processes. Being an early adopter of technology was not a priority or requirement for pilot users.

In our initial meetings with leadership, we had these champions—many of whom were themselves departmental leaders—share their experiences with the new tool. Their firsthand accounts were used to foster acceptance and enthusiasm, which leadership then carried forward to the wider organization. Our experience aligns with existing literature emphasizing the value of stakeholder engagement in successful implementations.^10^

### Low bar for entry

To encourage uptake of ambient scribing, we focused on developing a streamlined and flexible approach to training. Clinicians could visit a central hub website with clear, step-by-step instructions. While multiple options for live or on-demand webinars and one-on-one training were available, attendance was not mandatory, and live webinars were sparsely attended. Combined with the accessibility afforded by an enterprise-wide license and excitement generated by feedback from pilot users, this low-barrier approach contributed to adoption across the institution.

### Feasibility of simultaneous deployment

Following the pilot, we considered various strategies for expanding access to ambient scribing, including both a phased, incremental expansion across different clinics and a simultaneous enterprise-wide deployment. A phased rollout would allow for focused change management, continuous improvement with a smaller group, and a manageable load for technical and support teams but risked losing momentum and delaying full benefit. In contrast, a simultaneous rollout offered to thousands of providers would have the benefits of a cohesive enterprise-wide communication and support strategy, broad access, and rapid value realization but could risk overwhelming support infrastructure and amplifying the impact of any technical issues. Given the potential benefits of ambient scribing—along with no added cost per user under our enterprise license—we chose a “big bang” rollout. Overall, large-scale deployment was operationally feasible: support needs remained modest, most clinicians trained independently, and no safety events were reported.

### Understanding limited utilization

By the end of the study period, about half of users given access to ambient scribing had used the tool, and 20.1% of all visit notes were written using ambient scribing. Through direct feedback to our team, survey responses, and information gathered from on-the-ground “rounding blitzes,” we identified reasons for limited use of ambient scribing (Table 1).

**Table 1.** Reasons for limited use of ambient scribing.

Reasons for not trying ambient scribing	Reasons for incomplete or discontinued use of ambient scribing
Android user or lacking access to iOS device Unaware that the tool is available Lacking time needed to set up tool Feel that current workflow is already optimized Reluctant to use artificial intelligence for documentation	Editing drafted notes takes longer than existing workflow Currently use “copy-forward” to write notes which is not supported by ambient scribing Unhappy with style of drafted notes and lack of customization Drafted notes are too verbose Drafted notes contain errors Decided that the patient had too many complex problems to be accurately captured by ambient scribe (i.e., better for “single problem”) Ambient scribe did not capture nuances of informed consent discussion Patient/provider concerns about recording visit

Less than 100% adoption of the tool was likely related to a combination of these reasons, which reflect limitations of the technology itself in combination with our implementation strategy: By offering licenses to all clinicians, we reached a larger total number of users who might benefit from the technology, but with a larger denominator, the percentage using the tool was lower than it may have been with a smaller, more selective rollout. However, rather than aiming for 100% adoption, our goal was to offer ambient scribing to as many clinicians as possible as an available resource that could have a meaningful impact on documentation burden and clinician burnout, even if only a subset of clinicians chose to use it. As the tool matures, for instance, by improving customization, automating the pending of orders, and integrating clinical decision support, it may offer greater value to clinicians, leading to broader adoption.

### Limitations

This case report reflects the experience of a single academic health system and may not generalize to other settings. Surveys were sent only to clinicians who had used the tool at least once; non-users were not surveyed. Survey findings are limited to a 22.1% response rate among surveyed users and are subject to selection bias. Specifically, it is possible that users who responded to the survey were more likely to be enthusiastic about the technology, while non-respondents may have more often had a negative experience with the technology. We did not assess objective measures of clinician well-being, efficiency, documentation quality, long-term outcomes, or clinical impact, and findings are based on early adoption during the first 3 months post-deployment.

## Conclusion

Simultaneous enterprise-wide deployment of EHR-integrated ambient scribe technology was feasible, with lower-than-expected demand for technical support. A streamlined self-service training model and equitable access through an enterprise license provided immediate access for clinicians.

## Data Availability

The data underlying this article cannot be shared publicly due to the presence of protected health information. However, deidentified or summarized data will be shared on reasonable request to the corresponding author.
